# Ancient Mitogenomes Reveal the Domestication and Distribution of Cattle During the Longshan Culture Period in North China

**DOI:** 10.3389/fgene.2021.759827

**Published:** 2021-11-23

**Authors:** Xing Zhang, Liu Yang, Lingyun Hou, Hua Li, Hai Xiang, Xingbo Zhao

**Affiliations:** ^1^ National Engineering Laboratory for Animal Breeding, Key Laboratory of Animal Genetics, Breeding and Reproduction, Ministry of Agriculture, College of Animal Science and Technology, China Agricultural University, Beijing, China; ^2^ Guangdong Provincial Key Laboratory of Animal Molecular Design and Precise Breeding, School of Life Science and Engineering, Foshan University, Foshan, China

**Keywords:** *Bos taurus*, Ancient DNA, mitochondrial genome, domestication, longshan culture

## Abstract

Cattle, as an important tool for agricultural production in ancient China, have a complex history of domestication and distribution in China. Although it is generally accepted that ancient Chinese taurine cattle originated from the Near East, the explanation regarding their spread through China and whether or not this spread was associated with native aurochs during ancient times are still unclear. In this study, we obtained three nearly complete mitochondrial genomes (mitogenomes) from bovine remains dating back ca. 4,000 years at the Taosi and Guchengzhai sites in North China. For the first time at the mitogenome level, phylogenetic analyses confirmed the approximately 4,000-year-old bovines from North China as taurine cattle. All ancient cattle from both sites belonged to the T3 haplogroup, suggesting their origin from the Near East. The high affinity between ancient samples and southern Chinese taurine cattle indicated that ancient Chinese cattle had a genetic contribution to the taurine cattle of South China. A rapid decrease in the female effective population size ca. 4.65 thousand years ago (kya) and a steep increase ca. 1.99 kya occurred in Chinese taurine cattle. Overall, these results provide increasing evidence of the origin of cattle in the middle Yellow River region of China.

## Introduction

As one of the earliest domesticated animals (Diamond, 2002), cattle not only provide meat, dairy products, and leather but also played an important role in cultivation and other agricultural activities in the past 13,000 years (Ebersbach, 2002). According to their morphological characteristics and living habits, modern cattle are divided into humpless group (taurine, *Bos taurus*) and humped group (zebu, *Bos indicus*). Both archeological and genetic evidence have demonstrated that taurine cattle and zebu cattle were independently domesticated from aurochs in the Near East approximately 10,500 years before present (YBP) (Ajmone-Marsan et al., 2010) and in the Indus Valley approximately 8,500 YBP (Loftus et al., 1994), respectively, and then separately spread to the rest of the world following human migrations (Patel, 2009; Felius et al., 2014).

The abundant cattle resources in China, including the aurochs (*Bos primigenius*) existing in ancient China, as well as the zebu and taurine cattle in modern China, imply a complicated history of formation of Chinese taurine cattle (Qiu, 2006; Zhang et al., 2013; Cai et al., 2014; Brunson et al., 2016b). Analyses of partial mitochondrial DNA (mtDNA) sequences and complete mitochondrial genomes (mitogenomes) of various modern cattle breeds have revealed multiple region-specific maternal lineages [e.g., the T4 haplogroup was found mainly in East Asia (Felius et al., 2014)]. Analyses of mtDNA D-loop sequences revealed that the northern Chinese breeds originated from taurine cattle, the southern breeds originated from zebu cattle, whereas the central Chinese cattle breeds were of hybrid origin (Zhang et al., 2015). Whole-genome sequencing has revealed three different ancestors for the East Asian cattle, including East Asian taurine ancestry, Eurasian taurine ancestry, and Chinese indicine ancestry (Chen et al., 2018). Further genome analyses suggested that at least two historical migration events occurred in Chinese cattle in northern China (Chen et al., 2018).

Although the analyses of these modern specimens based on partial and complete genomes provide reasonable assumption into the study of cattle history, a direct ancient DNA (aDNA) analysis focusing on the ancient time has been valued more highly as it can provide a wealth of direct and solid evidence on the origin and dispersal of cattle species. In recent decades, advances in high throughput sequencing makes breakthroughs in aDNA research, which still performed inadequately. Specifically, analyses of ancient agricultural animal samples including pig, cattle, sheep and dog etc. especially from China, where exists numerous archeological sites, provide significant value to represent the history of animals and their companions, human society.

The earliest known taurine cattle remains in China were found in the Houtaomuga site in Jilin Province 5,500–5,300 YBP (Cai et al., 2018) and supported a significant population expansion of taurine cattle that spread from Northeast Asia into China. Through the discontinuous ancient DNA analysis on ancient cattle remains in China till now, it is widely accepted that Chinese taurine cattle were originated from the Near East (Brunson et al., 2016a; Cai et al., 2018) with two routes: Northwest entrance route and Northeast entrance route. In addition, genomic study of ancient cattle samples from the Shimao site has confirmed that the domestic cattle present at the site are pure East Asian taurine cattle (Chen et al., 2018). Although there is growing evidence that the domestication of Chinese cattle was a non-native process, the increasing excavation of auroch remains has made the origin of Chinese cattle contentious. Particularly, the exist of haplogroup C auroch 10,000 years ago in Northeast China (Zhang et al., 2013) and the evidence of haplogroup (P1a) incorporated into domestic cattle of northeastern Asia (Mannen et al., 2020) make it necessary to determine the species and phylogenetic relationships among suspected bovine remains from different sites in China, as well as to define the role that aurochs play in the development of taurine cattle in China.

## Materials and Methods

### Samples

**FIGURE 1:**
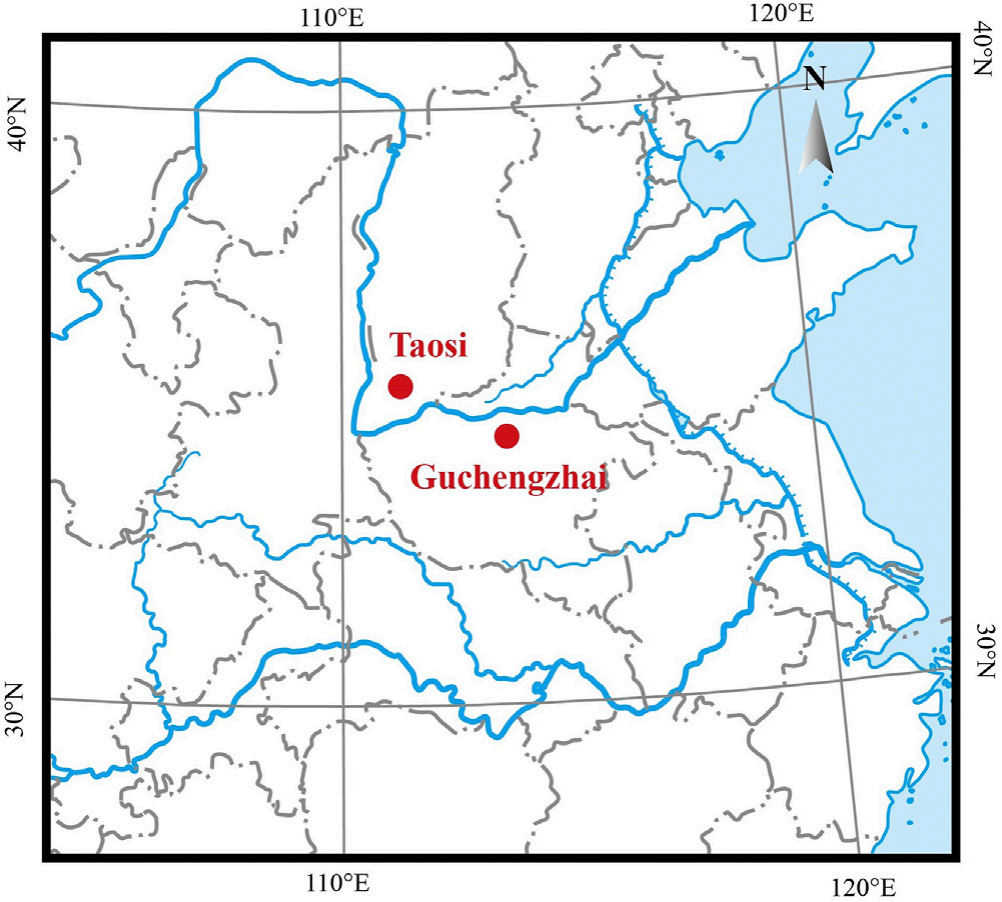
Location of the Taosi and Guchengzhai archaeological sites (red dots).

Bovine bone remains were collected from the Taosi and Guchengzhai sites. Both sites are located at the lower and middle reaches of the Yellow River drainage basin (Figure 1) and date back to the Longshan Culture period (approximately 4,000 YBP) (Supplementary Table S1). Specifically, the Taosi site is a large urban site located between the Ta’er Mountain and the Fen River, approximately 7.5 km northeast of Xiangfen County, Shanxi Province. The Taosi site dates back 4,350–3,900 years (Brunson et al., 2016a). The paleoenvironmental conditions around the Taosi site were warmer and wetter than now (Wang et al., 2011). Many domestic animals remain, including cattle bones, were found at the Taosi site; these animals were kept primarily as animals of the wealthy inhabitants (Brunson et al., 2016a). The Guchengzhai site is located in Dafanzhuang Village, 35 km southeast of Xinmi City, Henan Province, 1.5 km northeast of the intersection of the Zhenshui River and the Yan River. The Guchengzhai site dates back 4,150–3,950 years. Similarly, the paleoenvironment of the Guchengzhai site was slightly warmer and more humid than now (Xu et al., 2013). Domestic animals, including dogs, pigs, cattle, and sheep, as well as wild animals such as bears, sika deer, and elks were extensively discovered at the Guchengzhai site (Guo, 2018). The detailed information on all samples were shown in Supplementary Table S1. Before aDNA analysis, all samples were morphologically determined to be cattle remains.

### Extraction of Ancient DNA

All aDNA experiments were conducted in the Ancient DNA Laboratory at China Agricultural University and repeated independently in the Animal Genetic and Evolutionary Laboratory at Foshan University, following the experimental guidelines to eliminate contamination with modern samples. First, the equipment involved in the experiment was soaked with 5% (v/v) sodium hypochlorite solution and washed with 75% (vol/vol) alcohol, followed by UV irradiation for 1 h. Thereafter, the samples were prepared by cautiously removing the adhering soils on the surface of samples with a drill, followed by washing with 5% (vol/vol) sodium hypochlorite solution and then with double-distilled water and subsequent UV irradiation. The bones were then ground to fine-grained powder using an automatic sample quick grinding machine (Jingxin Industrial Development Co., Ltd., China), and 100–200 mg of powder was subjected to DNA extraction using a silica adsorption column system. At China Agricultural University, 50 μL of extracted DNA was obtained using the QIAamp DNA Investigator Kit (QIAGEN, Germany). At Foshan University, aDNA extraction was performed following the method described by Dabney (Dabney et al., 2013) with some adaptive modifications, including setting the lytic temperature to 45°C and extending the time of incubation with double distilled water (ddH_2_O) to 10 min.

### Mitogenome Capture and High-Throughput Sequencing

The mitogenomes of the genus *Bos*, including Holstein (*Bos taurus*), zebu (*Bos indicus*), gayal (*Bos frontalis*), and yak (*Bos grunniens*), were obtained using long-range PCR amplification. The PCR products were then used to prepare *Bos* hybrid baits. A total of 50 ng DNA per sample was used to construct a double-stranded DNA library using the KAPA Hyper Prep Kit for Illumina® platforms (KAPA Biosystems, San Francisco, USA) with minor modifications. The libraries were quantified using a Qubit 4.0 Fluorometer (Life Technologies Corporation, USA) and submitted for subsequent mitogenome enrichment and capture. Four libraries of Taosi samples were equally enriched to a total of 1 ng of DNA with unique adapter index sequences. Thereafter, mitogenome enrichment and hybrid capture were performed using the xGen Hybridization and Wash Kit (Integrated DNA Technologies, Inc., USA). The enriched library was then sequenced with 150 paired end reads (PE150) using the Illumina HiSeq X Ten platform. For the resequencing samples, up to 100 ng DNA per sample was used to prepare the library with the VAHTS Universal Plus DNA Library Prep Kit for MGI (Vazyme Biotech Co., Ltd, China). The qualified libraries were sequenced with PE100 using the MGI platform.

### Data Alignment, Filtering, and Authenticity Estimation

The quality of the raw data was assessed using the FastQC v0.11.9 (Andrews, 2010). Adapter and low-quality reads were filtered using clip&merge v1.7.8 from EAGER pipeline (Peltzer et al., 2016) with the following parameters: -*minlength* 25, --*minquality* 30. Before mapping, the first 250 and 500 bp of the taurine cattle reference mitogenome (GenBank accession No.: V00654) were concatenated to the end using CircularGenerate from the EAGER pipeline (Peltzer et al., 2016). The filtered reads were mapped to the circularized reference V00654 using CircularMapper v1.0 (Peltzer et al., 2016), with parameters as *-n* 0.01, *-l* 16,500. The resulting file was converted to a bam file and sorted using Samtools v1.9 (Li, 2011). Unmapped reads and reads with mapping quality <25 were removed using Samtools v1.9. Duplicate reads were removed using MarkDuplicates of Picard-tools v2.22.9 (http://broadinstitute.github.io/picard/). The endogenous DNA, coverage rate, and mapping quality distribution were calculated using Qualimap v2.2.2-dev (Okonechnikov et al., 2016). The authenticity of the retrieved sequences was evaluated according to the characteristics of the aDNA (Hofreiter et al., 2001; Willerslev and Cooper, 2005) using the mapDamage software (Jonsson et al., 2013).

### Assembly of Mitochondrial Genomes and Variant Calling

Three methods, including Mapping Iterative Assembler (MIAv.1.0) (Green et al., 2008), BCFtools (Li, 2011), and UnifiedGenotyper in the Genome Analysis Tool Kit (GATK, v3.5) (DePristo et al., 2011), were comparatively deployed to generate the mitogenome sequences. For MIA, the filtered bam files were converted to Fastq format using BEDtools (Quinlan and Hall, 2010). The program *ma* in MIA was used to generate complete sequences. The latter two software packages generate the complete sequence based on the mutation sites relative to the reference sequence using BCFtools consensus and manual generation, respectively. Finally, the complete mitogenome sequence of the ancient samples was obtained by comparing the differences in the complete sequences from different assembly methods and the filtered bam files.

Variant calling was carried out using BCFtools v1.8 mpileup (-d 5000 -Q 20 -a DP, AD, ADF, ADR, INFO/AD, INFO/ADF, INFO/ADR, SP, DV, DP4, DPR, INFO/DPR). Single nucleotide polymorphism (SNP) sites with <3× coverage depth were filtered out. UnifiedGenotyper in GATK3.5 was also used for the variation calling, whereas SNP sites with <3× coverage were discarded. Subsequently, the filtered SNP sites were manually checked and corrected according to the bam files visualized on the Integrative Genomics Viewer (IGV) (Thorvaldsdottir et al., 2013). Eventually, common SNP loci were retained as the true mutation loci.

### Sequence Alignment

All the captured and re-sequenced reads for the ancient samples were aligned to the reference mitogenome (GenBank accession No.: V00654) using the online MAFFT software with default parameters (Katoh et al., 2019). Positions embracing missing data or uncertain sites were removed using MEGA 7 (Kumar et al., 2016). Then the ratio of nonsynonymous and synonymous substitutions (dN/dS) was also calculated using MEGA 7. The 3 ancient consensus sequences were aligned to 36 others extant Bovinae mitogenomes from GenBank and called Dataset A (Supplementary Table S2), which included 1 *Bison,* 1 *Bison bonasus*, 1 *Bison schoetensacki*, 1 *Bison priscus*, 1 *Bubalus bubalis*, 1 *Bubalus depressicornis*, 1 *Bubalus carabanensis*, 1 *Bos frontalis*, 1 *Bos gaurus*, 1 *Bos grunniens*, 1 *Bos mutus*, 1 *Bos javanicus*, 3 *Bos primigenius,* 2 *Bos indicus*, and 19 *Bos taurus*. To further identify the haplogroup of the ancient samples, 510 cattle including *Bos taurus* and *Bos indicus* were aligned with ancient samples and called Dataset B (Supplementary Table S3).

### Phylogenetic and Demographic Analyses

To construct phylogenetic trees and infer population structure, the nucleotide substitution model was selected using jModelTest 2.1.1 (Darriba et al., 2012), based on the model selection strategy of Bayesian information criteria. Bayesian phylogenetic trees were constructed using MrBayes 3.2.7 (Ronquist et al., 2012), with the following parameters: 20,000,000 generations of Markov Chain Monte Carlo (MCMC) chains, checking every 10,000, sampling every 2,000, and discarding the first 10% as burn in. The consensus tree was constructed using FigTree v1.4.2 (http://tree.bio.ed.ac.uk/software/figtree/).

Principal component analysis (PCA) was used to further verify the genetic affinities of the ancient samples among the genus *Bos* using the R Package “adegenet” (Jombart, 2008). Part of dataset A was interrogated to infer demographics using BEAST 2.6.3 (Bouckaert et al., 2014). The ages of the ancient cattle were set at 4,000 YBP, which was the time of the culture in the respective sites of origin. We used Strict Clock as the clock model and Coalescent Bayesian Skyline as a distribution of the tree priors. The MCMC chains were set to 1000,000,000 generations. The log file was checked using Tracer v1.7.1 (Rambaut et al., 2018), and the Bayesian Skyline Plot (BSP) was obtained using Tracer v1.7.1. The optimal nucleotide substitution model was derived using MEGA 7 estimation for the maximum likelihood (ML) tree. Thereafter, an ML tree was built using MEGA 7 with 1,000 bootstrapping replicates and a timetree inferred using the Reltime method (Tamura et al., 2012). The timetree was computed using two calibration constraints from TimeTree (Kumar et al., 2017), including the time of divergence between *Bos taurus*, *Bos primigenius*, *Bos taurus*, and *Bos indicus.*

T haplogroups of Dataset B was used to calculate population pairwise *F*_*ST*_ values, the gene flow (Nm) and the net genetic distance using DNAsp 5.10 (Librado and Rozas, 2009). Meanwhile, numbers of haplotypes and variable sites, haplotype diversity (Hd), nucleotide diversity (Pi), and the average number of nucleotide differences (K) were estimated using the same data and software. Besides, T haplogroups of Dataset B was used to conduct PCoA (Principal Coordinate Analysis) among populations. The neighbor net (Bryant and Moulton, 2004) was constructed using splitstree5 (Huson and Bryant, 2006) based on *Fst* value. PCoA analysis was performed using the OmicStudio tools at https://www.omicstudio.cn/tool. The final result was graphed using R Package “ggplot2” (Hadley, 2016).

## Results

### aDNA Extraction and Sequencing

A total of eight ancient samples, including four bovines (4,350–3,850 YBP) from the Taosi site and four bovines (4,150–3,100 YBP) from the Guchengzhai site, were subjected to DNA extraction. All qualified aDNA extractions were selected for shotgun sequencing. However, owing to the typically low levels of endogenous DNA in these samples, only one sample from the Guchengzhai site (GCZ4C) obtained sufficient endogenous reads (0.0001%) to yield a near-complete mitogenome. Therefore, the remaining samples were subjected to mtDNA enrichment and hybridization sequencing using *Bos* hybrid baits. Total sequences of the near-complete mitogenome for the two samples from the Taosi site (TS1C and TS2C) were retrieved after enrichment using baits. After capture sequencing, the endogenous mtDNA read ratio increased to 0.3599% and 0.0474% for TS1C and TS2C, respectively (Table 1), resulting in the recovery of three complete ancient mitogenomes for late Neolithic archeological sites in the Middle Yellow River region. The average length of hybrid capture reads was approximately 140 bp, and the average length of resequencing reads was approximately 57 bp (Supplementary Figure S1). Damage pattern analyses showed a high C–T mutation at the 5′ end and a high G–A mutation at the 3′ end (Supplementary Figure S2), showing typical damage and fragmentation patterns characteristic of endogenous aDNA.

**TABLE 1:** Sequencing information of three ancient samples.

Sample name	RawBaseNum	FilBaseNum	mappedBaseNum	Duplication rate (%)	Endogenous mtDNA (%)	Mitogenome Length >1X	mtDNA coverage >1X (%)	Mean coverage	Mean length for mapped reads	Data sources
GCZ4C	162,165,074,800	87,622,422,828	57,698	5.50	0.0001	14,764	90.21	3.13	57.27 bp	resequencing
TS1C	57,079,200	31,114,331	111,968	19.80	0.3599	16,299	99.43	5.42	140 bp	capture
TS2C	210,187,800	116,303,356	55,160	21.40	0.0474	15,054	92.13	2.61	138 bp	capture

### Mitogenome Assembly of the Ancient Samples

By considering the effect on the integrity and accuracy of ancient mitogenome assembly, we combined three methods including GATK, BCFtools and MIA to generate mitogenome sequences. The number of duplicated sequences varied from 5.50% to 21.6% (Table 1). As a result, we obtained a 16,299 bp (coverage 99.43%) mitogenome sequence for TS1C, a 15,054 bp (coverage 92.13%) sequence for TS2C, and a 14,764 bp (coverage 90.21%) for GCZ4C (Table 1). SNP sites with ≥3× coverage were used in subsequent analyses (Supplementary Table S4).

### Species Identification

**FIGURE 2:**
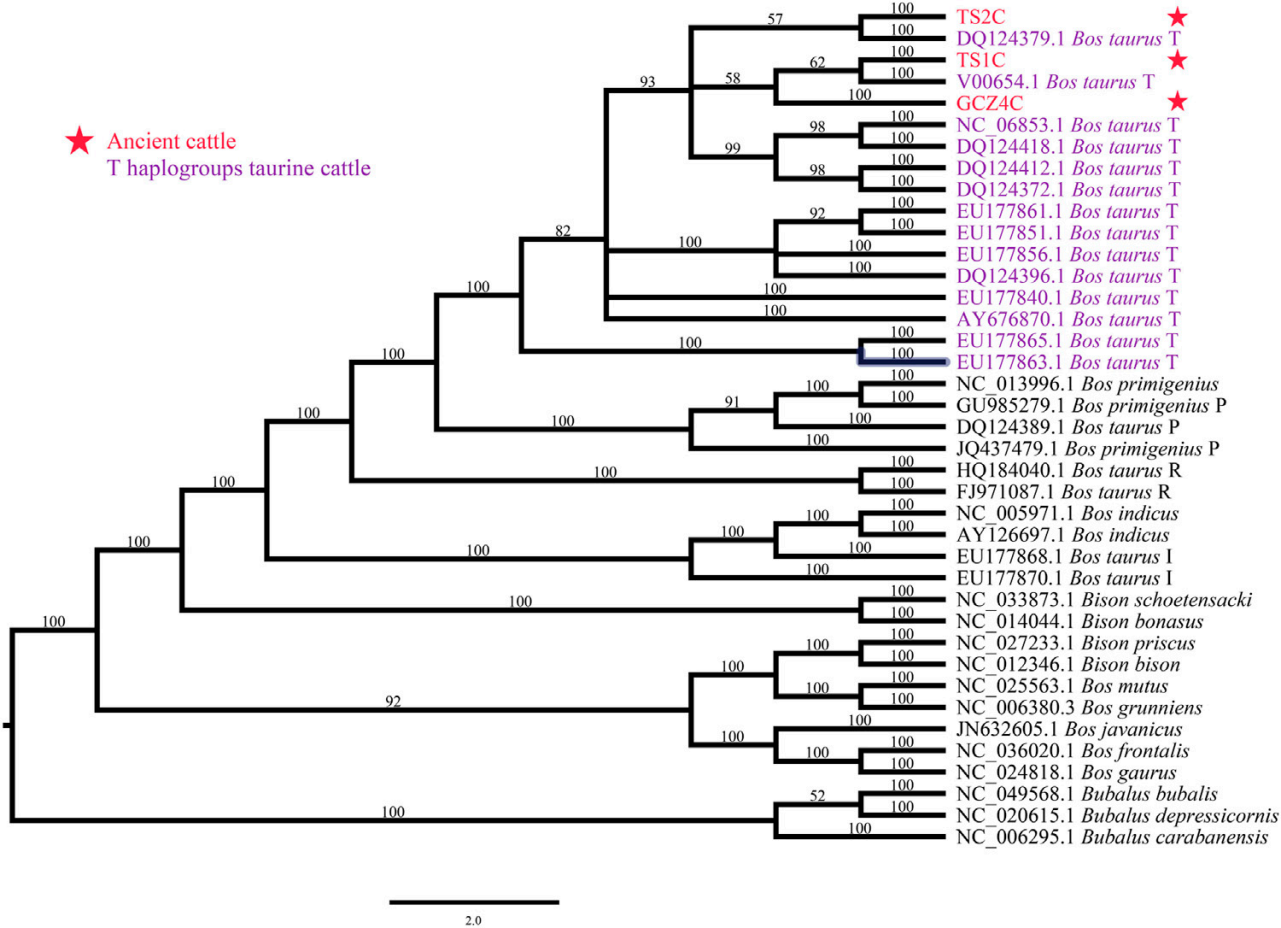
Species identification though phylogenetic analysis. ID marked with red font and star represents the ancient sample, ID with purple font represents T-haplogroup taurine cattle.

To determine the species of the ancient bovine samples, the near-complete mitogenome sequences were combined with 36 extant Bovinae mitogenomes, including the genus *Bison*, *Bubalus*, and *Bos* (Supplementary Table S2). The phylogenetic trees illustrated all three ancient samples belonging to the genus *Bos* rather than *Bison* or *Bubalus*, specifically close to the species *Bos taurus* (Figure 2 and Supplementary Figure S3).

### Phylogeny and Genetic Diversity of the Ancient Cattle

The mutation sites for each sample were discovered by alignment against the taurine cattle reference mitogenome (GenBank accession no.: V00654) (Supplementary Table S4). All ancient mitogenomes contained 13 coding genes, of which *COX3* had the most variable sites and the most non-synonymous SNPs. The values of dN/dS of all genes except *COX3* and *ND5* were lower than 1 (Supplementary Table S5), suggesting potential purifying selection on the mitochondrial functions in the ancient bovine in North China. Based on the key SNPs in the mitogenome, all three ancient samples were classified as haplogroup T3. Specifically, TS1C from the Taosi site belonged to haplotype T3k, while another sample, TS2C, from the Taosi site and GCZ4C from the Guchengzhai site belonged to haplotype T3n (Supplementary Table S4).

### Relationship of Ancient Cattle With Modern *Bos* Cattle

**FIGURE 3:**
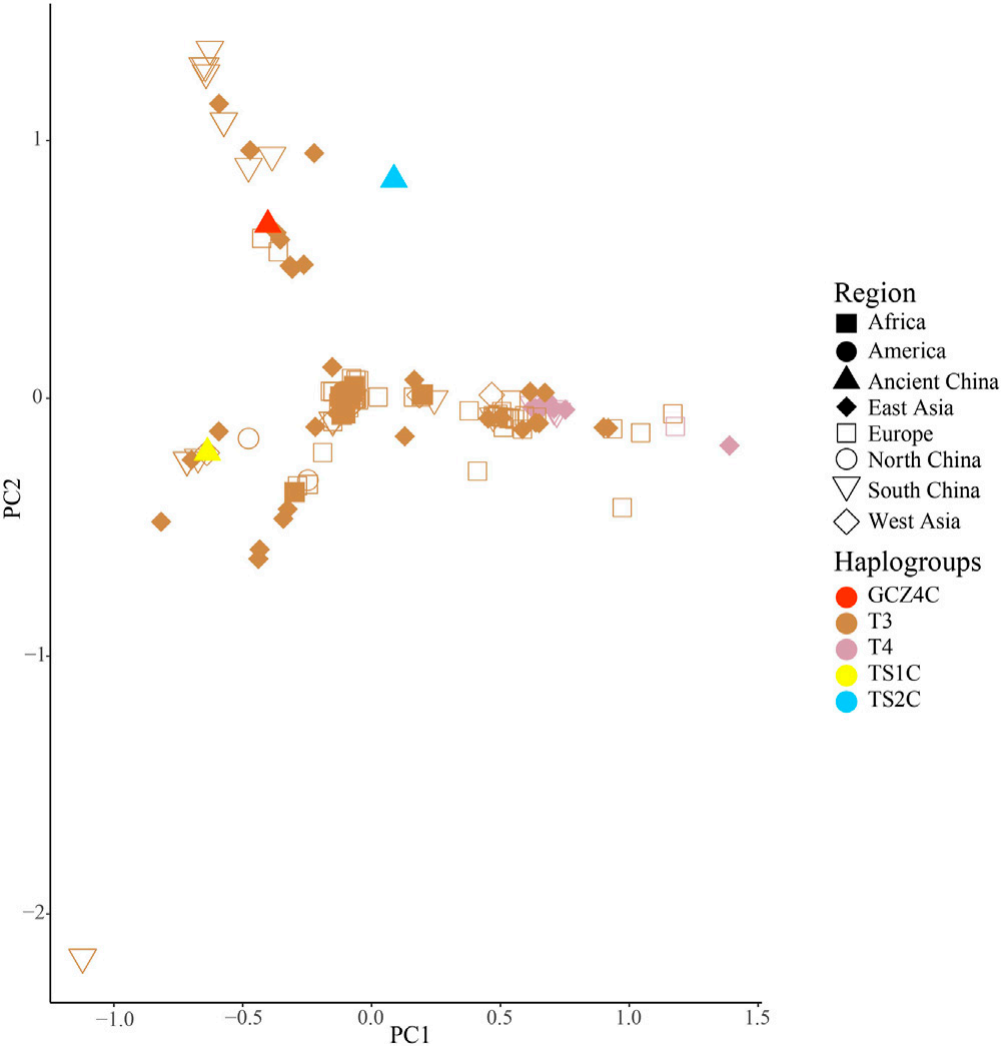
PCA analysis of ancient samples and T3/T4 species haplogroup *Bos*.

To further investigate the phylogenetic relationships among the approximately 4,000-year-old cattle in North China, a Bayesian phylogenetic tree was inferred with 510 extant mitogenome sequences of *Bos taurus* and *Bos indicus* around the world (Supplementary Table S3). The Bayesian tree showed that all ancient cattle were on the T3 branch (Supplementary Figure S4). PCA (Supplementary Figure S5A and S5B) and PCoA (Supplementary Figure S6A) supported the phylogeny of the T3 haplogroup of the ancient samples. The population pairwise *F*_*ST*_ values, the gene flow, and the net genetic distance, illustrated that the ancient Chinese cattle had the closest genetic relationship to Chinese taurine cattle, followed by populations from East Asia, Europe, West Asia, America, and Africa (Table 2). Further subdivision of Chinese taurine cattle according to region showed that population in T haplogroups had high haplotype and nucleotide diversity (Table 3), and the ancient Chinese cattle were more closely related to the south Chinese taurine cattle than the north taurine Chinese cattle (Table 2 and Supplementary Table S6). Moreover, PCA (Figure 3) and PCoA (Supplementary Figure S6B) analysis of ancient samples and T3/T4 cattle suggested that TS1C was genetically close to modern *Bos taurus* from Southern China, while TS2C and GCZ4C were closer to cattle from Europe and East Asia.

**TABLE 2:** The haplotype and nucleotide diversity of T haplogroup cattle in dataset B.

Group	Region	No. of samples	Variable sites	H	K	Hd ± SD	π ± SD
Ancient	Ancient	3	25	3	16.667	1.000 ± 0.272	0.00122 ± 0.00037
East Asia	East Asia	58	152	53	9.845	0.996 ± 0.004	0.00072 ± 0.00004
Africa	Africa	68	201	66	13.874	0.999 ± 0.003	0.00101 ± 0.00005
America	America	39	96	29	10.227	0.974 ± 0.014	0.00075 ± 0.00005
Europe	Europe	105	319	99	11.204	0.999 ± 0.002	0.00082 ± 0.00003
West Asia	West Asia	16	78	16	12.992	1.000 ± 0.022	0.00095 ± 0.00009
North China	North China	9	38	8	9.944	0.972 ± 0.064	0.00073 ± 0.00012
South China	South China	64	114	33	10.209	0.958 ± 0.014	0.00075 ± 0.00005
China	China	73	141	41	10.295	0.967 ± 0.011	0.00075 ± 0.00005

**TABLE 3:** Population pairwise *Fst* values (the below diagonal) and gene flow (Nm, the above diagonal) among T haplogroup of dataset B and ancient samples.

Region	Ancient	East Asia	Africa	America	Europe	West Asia	North China	South China	China
Ancient		3.96	1.66	3.44	3.80	4.30	4.92	7.70	7.69
East Asia	0.1120		1.69	4.95	8.49	7.98	16.20	7.65	9.02
Africa	0.2310	0.2279		4.28	3.33	2.88	1.85	2.85	2.75
America	0.1269	0.0918	0.1047		16.68	8.82	5.99	11.04	11.22
Europe	0.1164	0.0556	0.1307	0.0291		22.25	10.52	12.16	13.60
West Asia	0.1042	0.0590	0.1480	0.0537	0.0220		12.98	12.37	14.12
North china	0.0922	0.0299	0.2130	0.0770	0.0454	0.0371		10.23	20.97
South China	0.0610	0.0613	0.1495	0.0433	0.0395	0.0389	0.0466		−38.50
China	0.0611	0.0525	0.1540	0.0427	0.0355	0.0342	0.0233	−0.0132	

### Demographic Inferences

**FIGURE 4:**
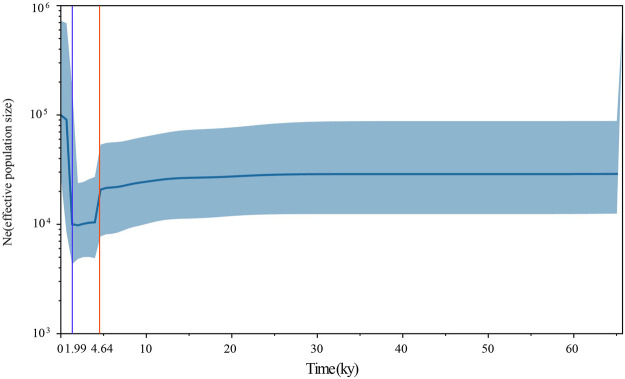
The Bayesian Skyline Plot of ancient and modern *Bos*. The Y-axis represents the effective number of populations, and the *X*-axis represents the timeline. The orange line shows the timepoint of effective population decline and the blue line shows the timepoint of the rapid increase of effective population.

**FIGURE 5:**
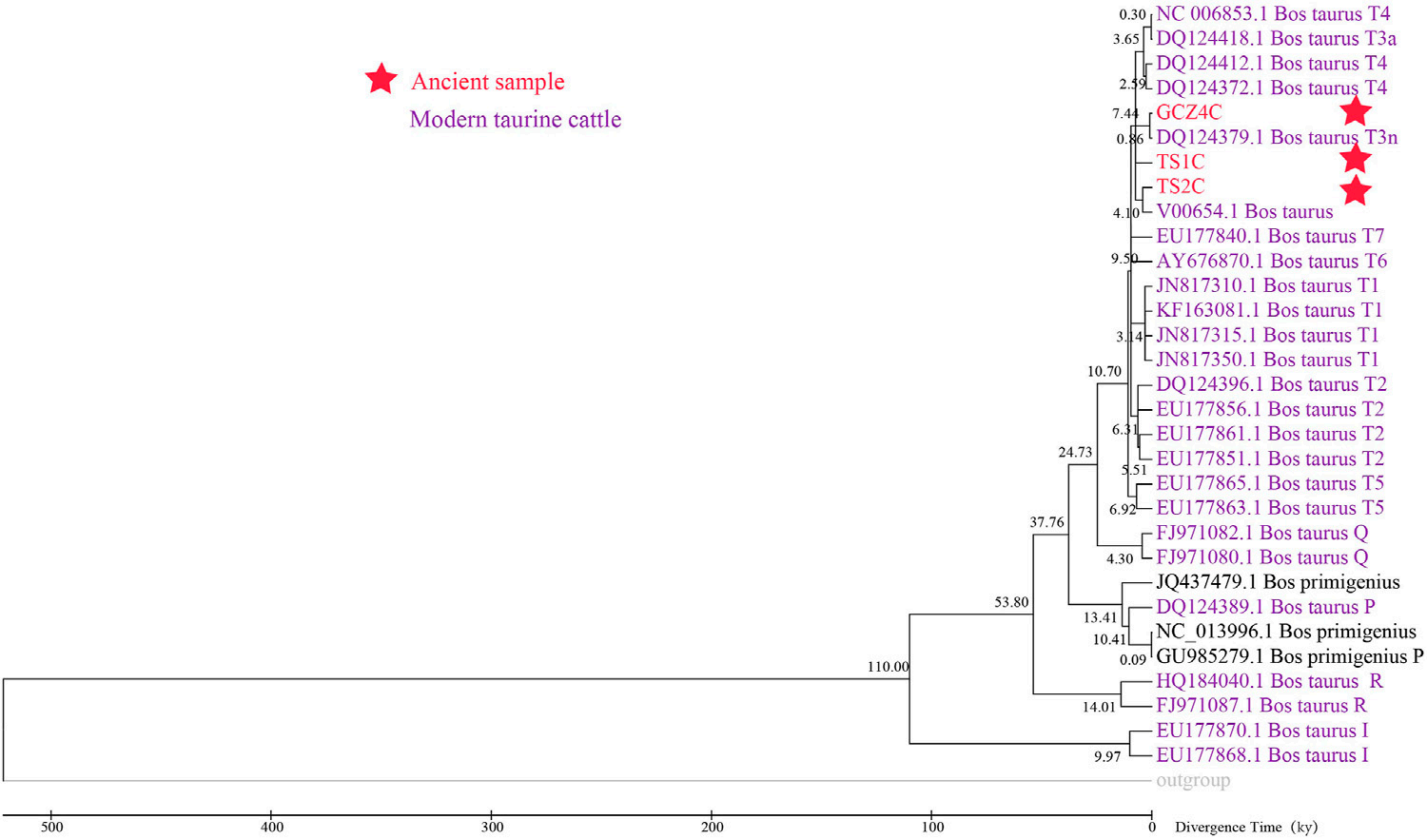
The Maximum Likelihood timetree of ancient and modern *Bos*.

To assess the population dynamics of taurine cattle in China, a BSP was produced using 25 mitogenomes. The BSP points to a rapid decrease in the female effective population size approximately 4.65 kya and a steep increase in population approximately 1.99 kya (Figure 4). To infer the divergence time between ancient and modern cattle, a ML tree was constructed with 3 *Bos primigenius* and 25 *Bos taurus* cattle, using 1 *Bison bonasus* as the outgroup (Figure 5). Concerning the differentiation of the major branches in the phylogenetic tree, the T3 and T4 sub-lineages possibly emerged approximately 7.44 kya, and at this time, the haplotype of sample TS1C appeared among the early domesticated cattle. TS2C, another sample from the Taosi site, was divergent to modern populations approximately 4.10 kya, in accordance with the period of rapid decrease in the female effective population size. GCZ4C was divergent to the modern T3n taurine population approximately 0.86 kya, suggesting possible long-term dilution of the ancient northern Chinese cattle.

## Discussion

Cattle genome research suggests that the Chinese taurine cattle originated from the Near East (Cai et al., 2014; Cai et al., 2018; Verdugo et al., 2019). According to the specific region where cattle remains were unearthed in China, it is believed that the earliest domesticated cattle in China occurred in northwestern China approximately 5,000–4,000 YBP (Flad et al., 2009). In addition, aDNA analysis of ancient Chinese cattle remains indicated that the cattle in northern China were taurine cattle since the Bronze Age (Cai et al., 2014). However, the abundant auroch remains prior to 5,000 YBP recovered from extensive regions in China suggest a potential early utilization and native domestication of Chinese bovines in the early Neolithic period (Zhang et al., 2013). Evidence from aDNA analysis of indigenous aurochs in central and northeast China during the Neolithic period adds to the uncertainty of the origins of the Chinese taurine cattle (Brunson et al., 2016b).

In this study, we obtained three nearly complete ancient mitogenomes from two ∼4,000 YBP archeological sites in North China and, for the first time, reported the complete mitogenomes from ancient bovines in this region. By determining all ancient samples as taurine cattle, no zebu population was found in these sites, providing further evidence that zebu cattle did not spread to northern China 4,000 YBP (Yue et al., 2014).

As one of the representative Longshan culture sites in China, the Taosi site is unique in that it contains numerous animal remains, such as pig mandibles, which proved that the ancestors living at the Taosi site mastered advanced animal husbandry technologies and lived a long-term, settled agricultural life (Nu, 2013). Different analyses of animal remains at the Taosi site help to understand the relationship between humans and animals under agricultural development in China. Isotopic analysis of the bones of pigs, cattle, and dogs indicated that the staple food of cattle at the Taosi site was a by-product of millet (Chen et al., 2012). Meanwhile, strontium isotope analysis indicated a mix of local and non-local animals at the Taosi site (Zhao et al., 2011). Additionally, the cattle were used for a variety of craft products at the Taosi site (Brunson et al., 2016a).

In our study, we analyzed the domestication status and genetic diversity of Chinese taurine cattle at the Taosi site at the level of the ancient mitogenome, which could be more accurate and direct than modern specimens. Both PCA, PCoA and phylogenetic analysis demonstrated that the ancient individuals of the Taosi site belonged to the T3 haplogroup, suggesting their origin from the Near East (Achilli et al., 2008), which was consistent with studies on mtDNA fragments (Cai et al., 2014; Brunson et al., 2016b). As shown, TS2C was localized to haplogroup T3n from East Asia, defined by 16119C, which probably had a founder effect (Cai et al., 2014). These data indicated that the appearance of ancient domestic cattle in northern China predated the Taosi site. A previous study based on a 294 bp mtDNA sequence showed that 15 ancient cattle in the Taosi site constituted 5 haplotypes (Cai et al., 2014). In our study, TS1C and TS2C originated from the same archeological site or even the same trench, but their haplotypes were different. This result reveals a high level of genetic diversity in the early cattle population at the Taosi site. TS1C diverged from the T3 haplogroup approximately ∼7.44 kya, while TS2C separated from the T3 haplogroup ∼4.10 kya, suggesting a rich diversity of cattle and the mixed presence of native and non-native cattle at the Taosi site.

The Guchengzhai site, dating back to the late period of the third Wangwan culture from Longshan culture in Henan Province, is one of the best-preserved ancient cities (Cai, 2002). A large number of wine vessels and animal remains, including pigs, cattle, and sheep have been found (Cai, 2008; Xu et al., 2013), indicating a locally developed agricultural level. However, genetic analysis of cattle remains at the Guchengzhai site has not been conducted. Through aDNA analysis, we generated the first complete mitogenome for Guchengzhai cattle and found that the bovine remains from this site belonged to taurine cattle. Interestingly, GCZ4C carries the same haplogroup (T3) as TS2C from the Taosi site, which has dominating founder effect in East Asian domestic cattle. Therefore, the similar genetic background shared by ancient cattle at Taosi and Guchengzhai site may indicate the early arrival of cattle at the northern China before the Longshan Culture period.

The *F*_*ST*_ values showed that ancient Chinese cattle have the closest affinity to modern Chinese cattle, indicating possible genetic contribution of ancient Chinese cattle populations to modern populations. Previously, whole-genome analysis of an ancient cattle from the Shimao site indicated that there were at least two migration events that occurred in Northern China (Chen et al., 2018). The closer genetic relationship of ancient Chinese cattle with the southern Chinese taurine than with the northwest China taurine may suggest that the ancient samples had genetic contributions to modern southern Chinese taurine cattle, and the northern taurine cattle may have been replaced by other taurine cattle. The BSP in our study also showed that the effective population size of cattle began to decline sharply approximately 4,500 YBP, in line with the arrival of taurine cattle into China. We also found that the ancient taurine cattle (TS2C) were separated approximately 4,000 YBP, which is a crucial period in the study of animal domestication in ancient China (Flad et al., 2009; Zhang et al., 2013; Brunson et al., 2016b; Cai et al., 2018). Meanwhile, the rapid increase in the effective cattle population approximately 1,900 YBP was also linked to the acceleration of communication between East and West at that time. During this period, the Han Empire in ancient China established the Silk Road to communicate with Western civilization. This information also suggests that cattle may have entered China *via* the Silk Road, increasing the effective population size of the Chinese cattle population.

## Conclusion

In this study, we for the first time obtained three near-complete mitogenomes of cattle about 4,000 YBP from North China and confirmed the ∼4,000-year-old bovine from North China as taurine cattle. All ancient cattle from Taosi and Guchengzhai sites belonged to the T3 haplogroup, suggesting their origin from Near East. The high affinity between ancient samples and southern Chinese taurine cattle indicated that the ancient Chinese cattle had a genetic contribution to taurine cattle of South China. A rapid decrease in the female effective population size ca. 4.65 kya and a steep increase ca. 1.99 kya occurred in Chinese taurine cattle.

## Data Availability

The original contributions presented in the study are publicly available in the China National GeneBank under accession number CNP0002141.

## Supplementary Material

Supplementary Figure S1 Distribution of fragment lengths of ancient DNA from three ancient samples.

Supplementary Figure S2 The DNA damage patterns of the ancient samples. Misincorporations frequencies are represented by the first and last 25 nucleotides of reads aligned to the bovine mitochondrial reference genome V00654.

Supplementary Figure S3 Species identification by phylogenetic analysis of three ancient samples with 36 extant Bovinae mitogenomes, respectively.

Supplementary Figure S4 Phylogenetic tree of three ancient mitogenomes and 510 cattle of Bos genus.

Supplementary Figure S5 PCA of ancient samples and species from the *Bos* genus. **(A)** shows the relationship of three ancient samples to all *Bos* genus while **(B)** shows their relationship to the T haplogroup *Bos* genus.

Supplementary Figure S6 PCoA of ancient samples and species from the *Bos* genus. **(A)** shows the relationship of three ancient samples to the T haplogroup *Bos* genus while **(B)** shows their relationship to the T3/T4 species haplogroup *Bos*.
