# Supplementary figures and images for: Ancient Mitogenomes Reveal the Domestication and Distribution of Cattle During the Longshan Culture Period in North China
Source: Front Genet. 2021 Nov 23;12:759827. doi: 10.3389/fgene.2021.759827 (PMC8650136; doi:10.3389/fgene.2021.759827)

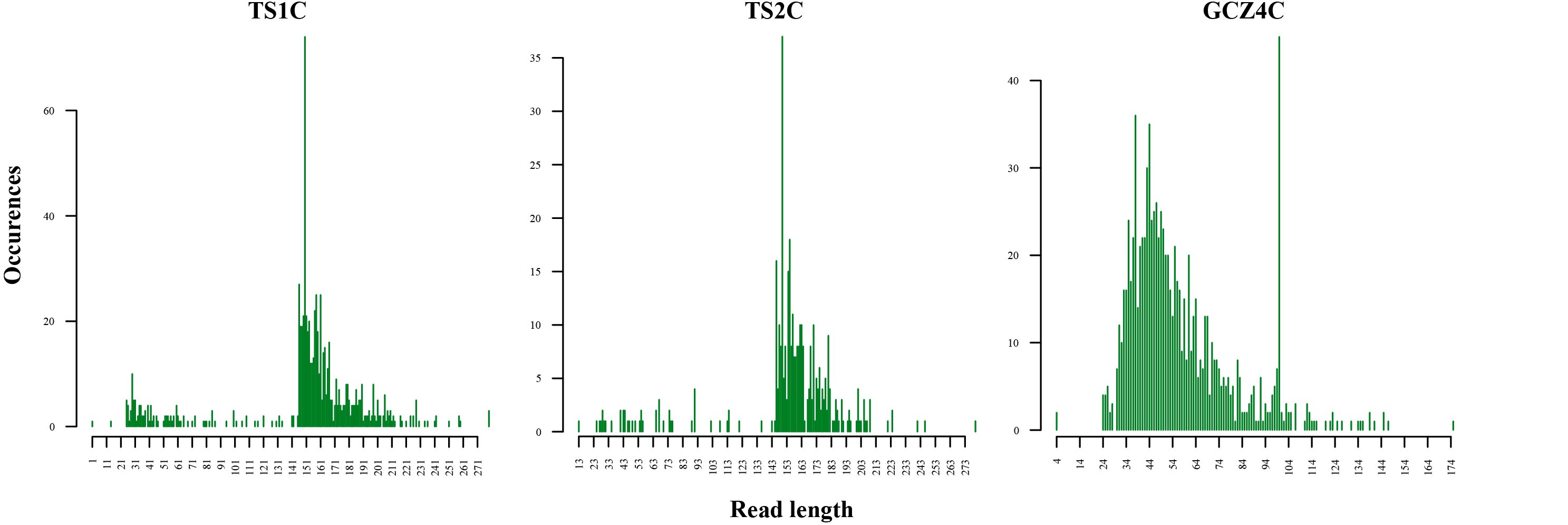

Supplement: Supplementary file 1 [file DataSheet2.zip › Figure 1.JPEG]

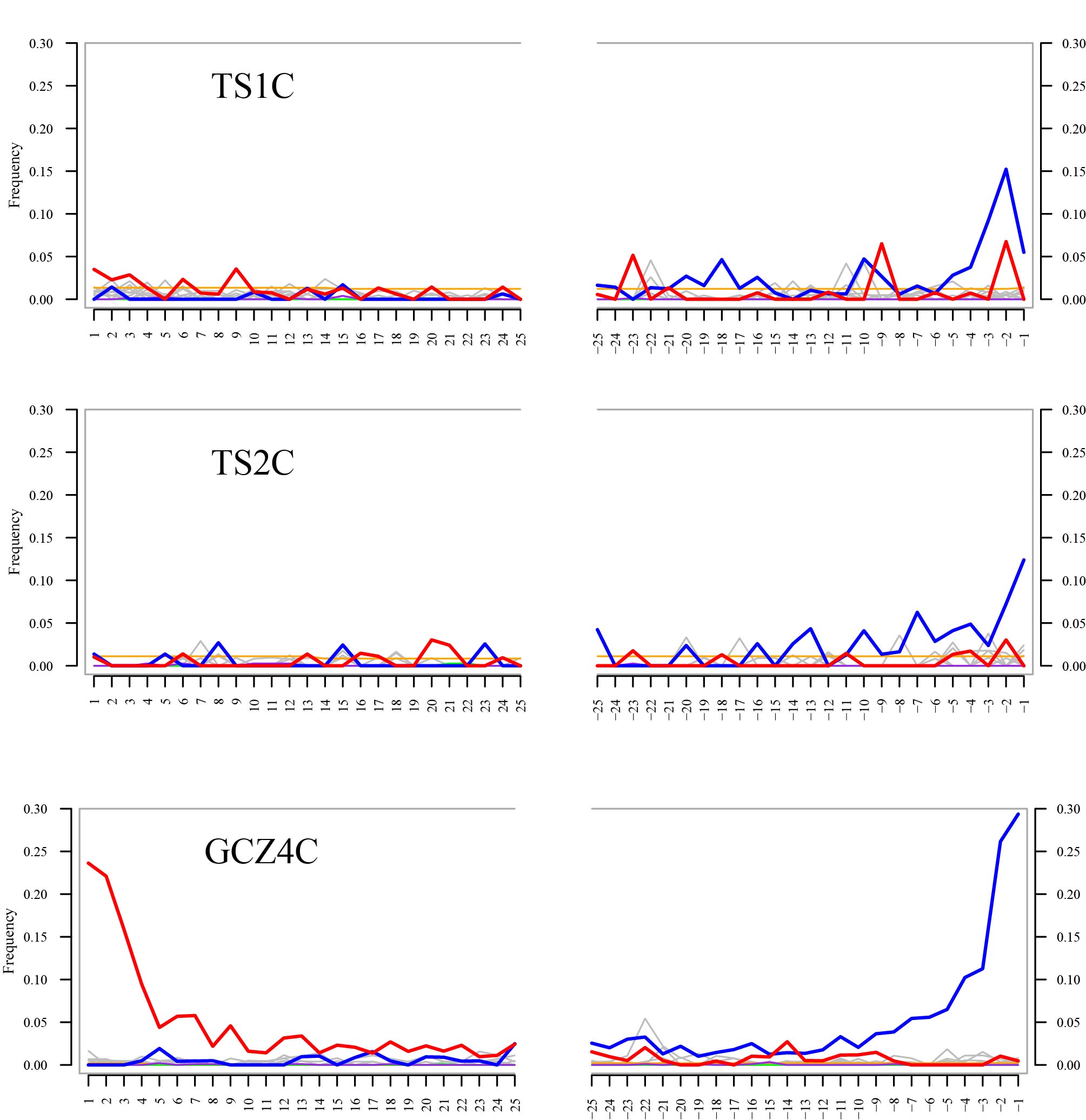

Supplement: Supplementary file 1 [file DataSheet2.zip › Figure 2.JPEG]

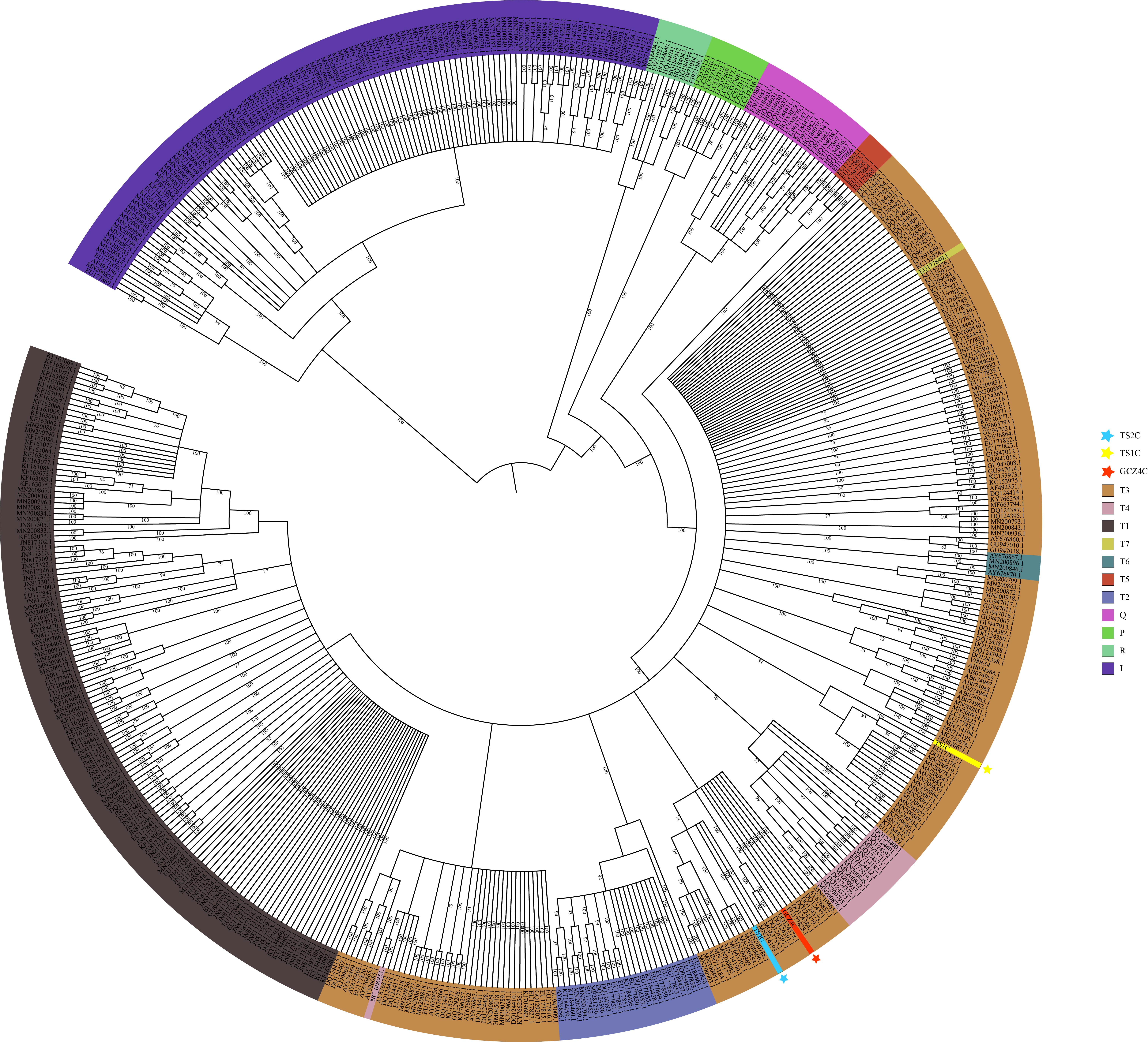

Supplement: Supplementary file 1 [file DataSheet2.zip › Figure 4.JPEG]

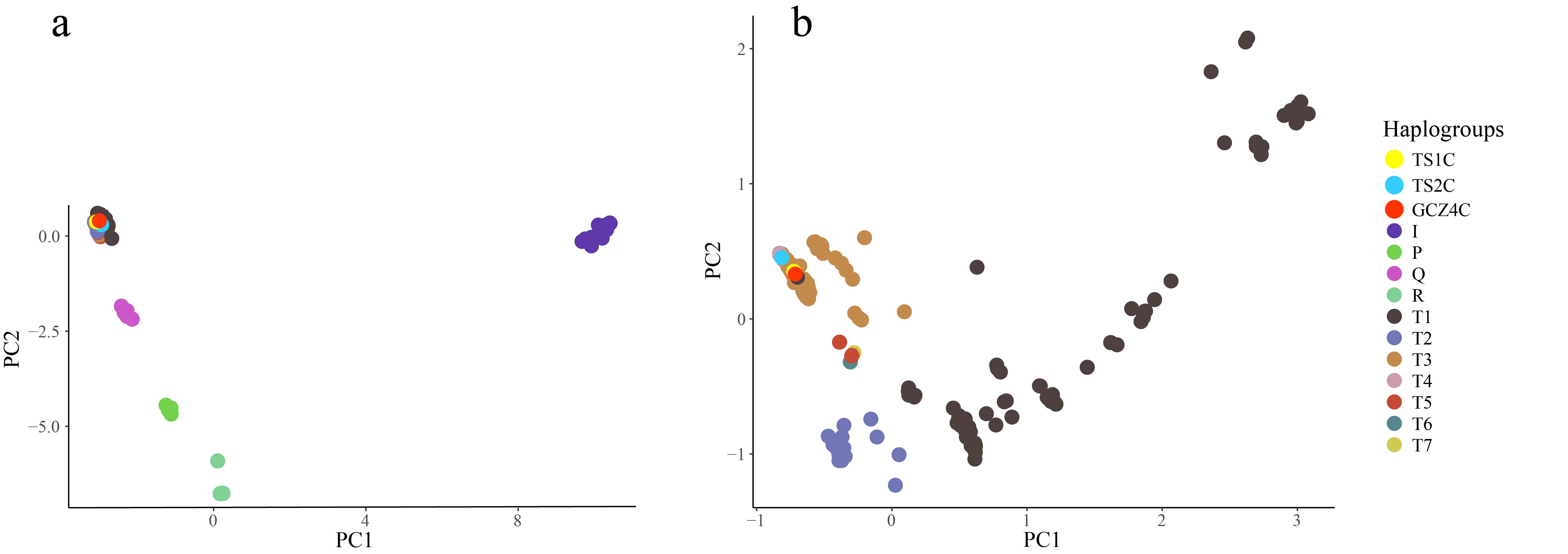

Supplement: Supplementary file 1 [file DataSheet2.zip › Figure 5.JPEG]

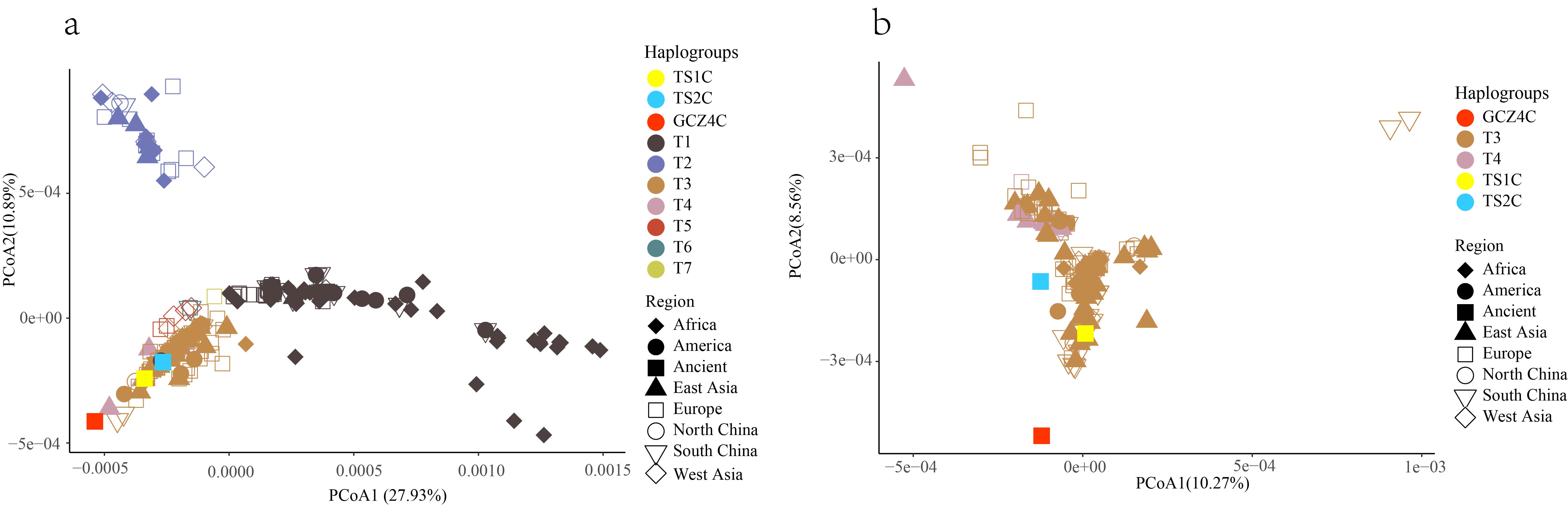

Supplement: Supplementary file 1 [file DataSheet2.zip › Figure 6.JPEG]
